# The interplay between microRNAs and Nrf2 signaling in human cancers

**DOI:** 10.1186/s12935-024-03430-1

**Published:** 2024-07-05

**Authors:** Reza Panahizadeh, Mohammad Amin Vatankhah, Ali Safari, Hesam Danesh, Negin Nazmi, Pourya Gholizadeh, Narges Soozangar, Farhad Jeddi

**Affiliations:** 1 Zoonoses Research Center, Ardabil University of Medical Sciences, Ardabil, Iran; 2 Department of Orthopedics, Shohada Hospital, Tabriz University of Medical Sciences, Tabriz, Iran; 3 School of Medicine, Islamic Azad University, Ardabil, Iran; 4 Digestive Disease Research Center, Ardabil University of Medical Sciences, Ardabil, Iran; 5 Department of Genetics and Pathology, School of Medicine, Ardabil University of Medical Sciences, Ardabil, Iran

**Keywords:** miRNAs, Cancer, Keap1, Nrf2

## Abstract

MicroRNAs (miRNAs), as a class of nonprotein-coding RNAs, post-transcriptionally regulate the expression of target genes by base pairing to 3’-untranslated regions (3'‐UTRs). Nuclear factor E2-related factor 2 (Nrf2) has been identified as a critical component of the antioxidant defense mechanism. Dysregulation is associated with chemoresistance and radioresistance in cancerous cells. MiRNA-mediated regulation of the Nrf2 signaling pathway has been shown to have important implications for the development of various cancers. In this article, we review the roles of miRNAs as regulators of the Nrf2 pathway in different human cancers. Ras‐associated binding (Rab) proteins have an essential role regulation of vesicle transport, as well as oncogenic functions in preventing chemotherapy efficacy and cancer development. More importantly, increased evidence indicated that the interaction between miRNAs and Rabs has been determined to play critical roles in cancer therapy. However, the significant limitations in using miRNAs for therapeutic applications include cross‐targeting and instability of miRNAs. The detailed aspect of the interaction of miRNAs and Rabs is not clearly understood. In the current review, we highlighted the involvement of these molecules as regulators of the Nrf2 pathway in cancer pathogenesis. Potential methods and several obstacles in developing miRNAs as an anticancer therapy are also mentioned.

## Introduction

The incidence of human cancer is rising globally due to economic growth, aging, and globalization. Despite the excellent survival rate brought about by advances in diagnostic and therapeutic techniques, the burden of cancer is continually increasing. In 2040, there will likely be 28.4 million new cancer cases worldwide, meaning a 47% increase related to 2020. The only hope we now have for controlling cancer is a deeper understanding of the molecular basis of the disease [1].

Nrf2 is a crucial component of the antioxidant defense mechanism. The expressions of Nrf2 proteins are normally kept at deficient levels, but oxidative stressors cause them to build up and become transactivated. Through suppression of cancer cell apoptosis and improvement of the ability of cancer stem cells to self-renew, constitutive activation of Nrf2 in many malignancies enhances cancer cell proliferation [2]. More significantly, it has been shown that Nrf2 promotes the chemoresistance and the radioresistance of cancerous cells. This indicates the promising potential of Nrf2 inhibition as an attempt to treat cancer [3, 4].

Several efforts have been made to affect the Nrf2 signaling pathway to prevent and treat cancer. Most MicroRNAs (miRNAs) hybridize with the 3’ untranslated region (3’UTR) of target mRNAs in a sequence-specific manner and thereby block the translation of mRNAs in ribosomes and/or facilitate the mRNA degradation. In recent years, there has been increasing evidence that miRNAs also bind in the coding region (CDS) and 5’UTR, but the implication of these interactions remains obscure because they have a smaller impact on mRNA stability compared with miRNA-target interactions that involve 3’ UTRs. Finally, different families of miRNAs that are broadly expressed but are active in different contexts show distinct preferences for the CDS, 3' UTR, and 5’UTR [5, 6].

**Fig. 1:**
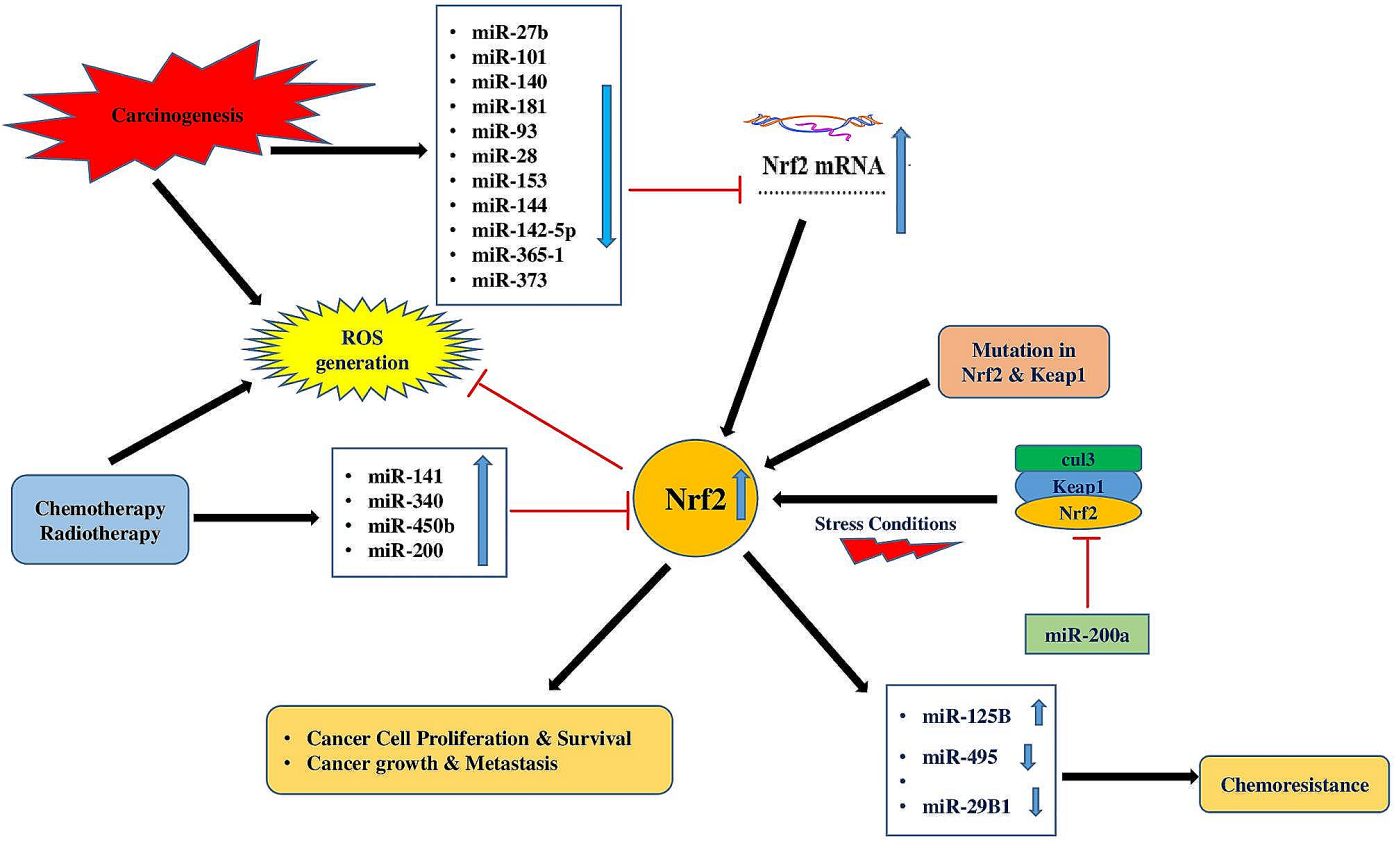
Representation of the intricate interplay between Nrf2, microRNAs, several signaling pathways and ROS generation in mediating chemoresistance and radioresistance in cancer cells: This schematic illustrates the pivotal role of Nrf2 in mediating chemoresistance and radioresistance through its interactions with various MicroRNAs, which modulate ROS generation. The intricate network depicted elucidates the interconnected pathways through which Nrf2 and MicroRNAs contribute to the adaptive responses of cancer cells to chemotherapy and radiotherapy. By showcasing the regulatory crosstalk among Nrf2, MicroRNAs, and ROS generation in the context of resistance mechanisms, this figure provides valuable insights into potential therapeutic targets for overcoming treatment resistance in cancer

MicroRNAs can influence the *3' UTR* of Nrf2 mRNA at different stages, making them one of the potential upstream modulators of the Nrf2 signaling pathway. The Nrf2 pathway has been demonstrated to be regulated by miRNAs via various key mechanisms, such as altering Nrf2 nuclear translocation, impacting Nrf2 expression, controlling Nrf2 upstream mediators, and modulating Kelch-like ECH-associated protein 1 (Keap1), as a negative regulator of Nrf2 (Fig. 1) [7–10]. Accumulating evidence suggests that miRNA-mediated regulation of the Nrf2 signaling pathway has important implications for developing various cancers [11, 12]. Here, we summarize the roles of miRNAs as regulators of the Nrf2 pathway in different human cancers.

## Esophageal cancer

Esophageal cancer ranks as the sixth leading cause of cancer-related deaths globally. In 2020, approximately 604,000 new cases were diagnosed worldwide, resulting in around 544,000 deaths [1]. ESCC accounts for nearly 90% of all esophageal cancer cases worldwide and is prevalent in Eastern and Central Asia, as well as in East and South Africa [13].

MiRNAs play critical roles in the pathogenesis of ESCC. Therefore, clarifying epigenetic changes in microRNA expression and molecular mechanisms involved in ESCC and identifying molecular targets will help improve the disease’s prevention, early diagnosis, management, and prognosis [14]. Han et al. conducted a recent study on miR-27b-3p expression in ESCC. They observed a significant downregulation of miR-27b-3p in ESCC tissues compared to adjacent tissues. Furthermore, lower miR-27b-3p expression correlated with TNM stage, poor cell differentiation, and lymph node metastasis. Functionally, miR-27b-3p inhibited cell proliferation, invasion, and migration in ESCC cells, with overexpression of miR-27b-3p leading to decreased Nrf2 protein expression. Additionally, higher Nrf2 mRNA expression in ESCC tissues was associated with advanced lymph node metastasis and TNM stage. Taken together, these findings confirmed the role of miR-27b-3p in suppressing ESCC progression through Nrf2 targeting [15]. In another study, Mei Liu et al. showed the impact of Methylseleninic acid (MSA) on the Keap1/Nrf2 pathway in ESCC cells. Treatment with MSA in both KYSE180 and KYSE150 cell lines led to decreased Keap1 and increased Nrf2 expression at the protein level. These findings suggested that Keap1 serves as a direct target of miR-200a in ESCC cells. Furthermore, MSA treatment induced miR-200a expression through KLF4 mediation. However, KLF4 knockdown prevented MSA-induced miR-200a expression, establishing a connection among KLF4, Keap1 downregulation, and Nrf2 upregulation by MSA [16]. These data support the notion that the chemopreventive property of MSA in esophageal carcinogenesis may depend on the regulation of the KLF4/miR-200a/Keap1/Nrf2 axis.

## Gastric cancer (GC)

GC is a significant health concern characterized by chemoresistance and poor overall survival in advanced stages. Epidemiological data confirm GC is the fifth most common cause of cancer and the fourth most frequent cause of cancer-related death worldwide. In 2020, over 1,089,000 new GC cases were diagnosed globally [1, 17, 18].

In cancer pathogenesis, abnormally expressed miRNAs can act as oncogenic miRNAs or tumor suppressors. Studies have shown that Nrf2 has the ability to regulate several miRNAs [19, 20]. Furthermore, numerous cancer–related miRNAs have been discovered to be upregulated or downregulated in GC [21, 22]. For example, Dong et al. identified dysregulation of cancer-related miRNAs in gastric cancer. Their study revealed that transfection with miR-101 mimics significantly increased apoptotic protein expression, including activated Caspase-3 and Bcl-2-associated X protein (Bax). MiR-101 notably reduced the proliferative abilities of GC cells and inhibited overall cell proliferation. In addition, miR-101 was found to interact with Nrf2 mRNAs via complementary sites in the 3’-UTR, suggesting a negative regulatory role of miR-101 on Nrf2. The miR-101/Nrf2 axis appears to play a crucial role in gastric mucosal epithelial cell apoptosis and proliferation. Thus, overexpressing miR-101 to downregulate Nrf2 holds promise as a therapeutic strategy for gastric cancer [23].

## Breast cancer

Breast cancer is one of the most preventable cancers, the leading cause of global cancer incidence, and the 5th reason for cancer-related death worldwide [1]. The most common type of breast cancer is invasive ductal carcinoma (in 50-75% of patients), and the preceding is invasive lobular carcinoma (in 5-15% of patients) [24].

Nrf2 increases phase II detoxifying and anti-oxidant enzymes such as glucuronosyl transferases, γ-glutamyl cysteine synthetase, NQO1, and GSTs via binding to anti-oxidant response elements [25, 26]. These roles of Nrf2 are associated with the interaction between Nrf2 and Keap1. According to recent studies, the miR-200 family plays vital functions in the proliferation of cancerous cells, especially breast cancer. Therefore, the targeting of the Nrf2/Keap1/miR-200 axis could be an appropriate choice for breast cancer treatment [27, 28]. Eades et al. illustrated that the expression of miR-200 is attenuated in breast cancer cells and dysregulation of miR-200 results in down-regulation of Nrf2 and Keap1 protein expression [29]. Suberoylanilide hydroxamic acid (SAHA) as a histone deacetylase (HDAC) inhibitor causes the re-expression of epigenetically silenced genes, and it can be used in the treatment of various neoplastic cells [30]. In another study, treatment of breast cancer cells with SAHA reduced proliferation rate through up-regulation of miR-200 Nrf2. Therefore, epigenetic treatment (HDAC inhibitors) can constrain breast cancer cell growth, in part, via activation of the Nrf2-dependent antioxidant pathways, mediated by the re-expression of miR-200a and the targeting of the Keap1 3’-UTR. [29].

As a vital member of the tumor microenvironment, hypoxia has crucial functions in the proliferation, migration, metastasis, and angiogenesis of cancerous cells [31]. Hif-1α is a prolyl hydroxylase domain-containing protein that regulates the response of cells to hypoxia through its interaction with ROS [32]. Hypoxia causes oxidative stress by producing ROS, and this process activates Nrf2, which regulates Hif-1α [33]. Previous studies indicated that hypoxia up-regulates Hif-1α and HO-1 expression through Nrf2 transition into the nucleus and targeting the PI3K/Akt signaling pathway [34]. Altogether, the examination of hypoxia mechanisms is critical because it represents a barrier to the cancer treatment approach. In 2021, Mahajan et al. revealed that hypoxic conditions attenuated miR-140 expression and enhanced the expression of HO-1 and Nrf2 in breast cancer cells. Conversely, up-regulation of miR-140 decreased the expression of Hif-1α under hypoxia conditions. They showed that miR-140 up-regulation inhibits colony formation and proliferation under hypoxia in breast cancer cells by reducing cell adhesion on collagen IV, laminin, and fibronectin. Over-expression of miR-140 suppresses breast cancer angiogenesis by decreasing the secretion of IL-8, VEGF, and Nrf2 [35].

Blasio et al. showed that the higher Nrf2 expression leads to the decreased expression of miR-29b‐1‐5p in MDA‐MB‐231 cells. Nrf2 decreases ROS generation, improving MDA‐MB‐231 cell proliferation, while miR‐29b‐1‐5p increases intracellular ROS, reducing cell growth rate. Nrf2 phosphorylation needs nuclear translocation and transcription activation that mediates with some protein kinases such as AKT. Also, miR‐29b‐1‐5p overexpression caused a significant decrease in p‐AKT levels. In contrast, Nrf2 activation raised the levels of p‐AKT. And, miR‐29b‐1‐5p suppresses the Nrf2 by regulating the AKT pathway. Overall, the present study reveals that NRF2 activation downregulates miR‐29b‐1‐5p, suggesting that NRF2 can also exert its protective effect by regulating miR-29b-1-5p [36].

The miR-101 overexpression could diminish the proliferation rate and colony formation of MCF-7 breast cancer cells. Also, miR-101 significantly raised the apoptosis rate of MCF-7 cells by downregulation of Nrf2 expression [37].

In another study, Lee et al. showed that silencing Nrf2 reduced ATP production and mitochondrial membrane potential (MMP) levels. Also, HIF-1α collection levels were significantly decreased in MDA-MB-231 and MCF-7 breast cancer cell lines. These findings suggest that Nrf2 pathway silencing suppresses the glycolysis pathway across hypoxia conditions through HIF-1α dysregulation. Also, it has been shown that miR-181c overexpression is a mediator for regulating, HIF-1α signaling in Nrf2-silenced breast cancer cell lines. Hypoxic cell lines might undergo catabolic procedures to recompense oxidative stress under hypoxic situations, and Nrf2-silencing suppresses hypoxia-mediated catabolism. The most crucial catabolic program under hypoxic circumstances is autophagy, and activation of HIF-1α-mediated autophagy is repressed in hypoxic Nrf2-silenced cells. Overexpression of miR-181c drives the impairment in activation of HIF-1α/BNIP3-mediated autophagy in Nrf2-silenced breast cancer cells [38].

Two-thirds of human breast cancers are estrogen-dependent, and oxidative stress plays an important role in estrogen-induced breast carcinogenesis [39, 40]. The functions of microRNA-93 and transcription factor Nrf2 in mammary cells have been investigated by Singh et al., to describe the molecular mechanism of estrogen-associated breast cancers. This investigation showed that estrogen (E2) treatment significantly increases miR-93 expression and downregulates Nrf2 expression in mammary tumors, rat mammary tissues, and human neoplastic breast epithelial cell line T47D and human normal (non-neoplastic) breast epithelial cell line MCF-10 A compared to respective controls. On the other hand, Vitamin C (as an antioxidant) inhibits the estrogen-mediated increase in miR-93 and upregulates Nrf2. So, there is an inverse association between Nrf2 protein expression and miR-93 expression. It also proved that antimiR-93-mediated silencing of miR-93 increases protein expression of Nrf2-regulated genes and Nrf2 in MCF-10 A and T47D cell lines. In contrast, transfection of premiR-93 decreases Nrf2 protein expression in MCF-10 A and T47D cells. Therefore, Nrf2 is a potential target of miR-93. MiR-93 increases the migratory properties, mammosphere, and clonability formation of MCF-10 A cells, contributing to their carcinogenic potential. MiR-93 inhibits apoptosis by decreasing caspase-3/7 activity and increases oxidative DNA damage in MCF-10 A cells compared to control cells [41].

Nrf2 is a transcription factor involved in antioxidant response. It promotes antioxidant enzyme expression and protects cells from carcinogen-induced DNA damage. Therefore, loss of Nrf2 contributes to carcinogenesis [42]. Nrf2 is regulated post-translationally by the Keap1 protein. It is also regulated at the transcription and translation levels. MiRNAs can inhibit gene expression by binding to the 3´UTR region of target mRNAs [43]. Yang et al. investigated the miR-28 effect on Nrf2 regulation in another study. MiR-28 and Nrf2 expression were assessed in the MCF-7 cell line and normal human mammary epithelial cells (HMEC). The results revealed higher levels of Nrf2 expression and lower levels of miR-28 in the MCF-7 cell line compared to HMEC. There was an inverse relationship between the expression pattern of miR-28 and Nrf2 in both cell lines. This study demonstrated that estrogen treatment increases Nrf2 mRNAs and decreases miR-28 levels in HMEC compared to control groups. MiR-28 negatively regulated Nrf2 expression by targeting 3´UTR of Nrf2 mRNA. It also decreased Nrf2 protein stability. Inhibition of Nrf2 expression by miR-28 was interestingly independent of Keap1 protein. This study also suggested that loss of Nrf2 can potentiate colony formation in the MCF-7 cell line [44].

## Acute myeloid leukemia (AML)

Nrf2 is a crucial regulator of oxidative stress conditions that protects cells from ROS by the regulation of several cytoprotective genes, including HO-1 and NQO1 [45]. Nrf2 contributes not only to the malignant phenotype of AML but also contributes to AML resistance to standard chemotherapy [46]. It has been shown that miRNAs expression may become dysregulated in some cancers, and they could be regulated by Nrf2 [47]. Shah et al. investigated the Nrf2 effects on miRNAs in human AML cells. Experiments on THP-1 cells (human leukemia monocytic cell line derived from AML) showed that enhanced Nrf2 activity declines miR-29B expression and increases miR-125B in AML. Further tests revealed that miR-125B1 and miR-29B1, but not miR-125B2 and miR-29B2 are regulated by Nrf2. This study demonstrated that Nrf2 binds to antioxidant response element (ARE) regions in the promoters of miR-125B1 and miR-29B1, especially the miR-125B1 ARE5 site and miR-29B1 ARE3 site. It has been shown that Nrf2 and miR-125B1 are increased in AML in comparison with normal CD34^+^ hematopoietic stem cells (HSC), while miR-29B1 is reduced in AML compared with normal CG34^+^ HSC [48]. Upregulation of miR-125B exerts an oncogenic role through downregulation of miR-29B, represses apoptosis, increases proliferation, and suppresses apoptosis [49]. AML with normal Nrf2 and normal miR-125B and miR-29B expression was much more sensitive to daunorubicin than AML patient samples that had high Nrf2 expression and thus high miR-125B and low miR-29B expression. Moreover, transfection of miR-125B antagomiR and miR-29B mimics the primary AML and increases the sensitivity of these AML cells to daunorubicin. miR-29B and MiR-125B synergistically affect chemoresistance and AML survival, probably by deregulating target genes including BAK1, AKT2, or STAT3. In conclusion, NRF2 regulation of miR-29B and miR-125B promotes the survival of cancer cells and their alteration increases the response to cytotoxic chemotherapy in AML [48].

## Multiple myeloma (MM)

MM is an infrequent blood disease, responsible for 1% of cancers and 10% of all hematological malignancies, and is the second most common blood cancer [50]. MM is known for bone marrow (BM) infiltration of monoclonal plasma cells (PC) that secrete monoclonal antibodies that can be found in the blood or urine [51].

Dysregulation of miRNAs expression has been shown in Multiple Myeloma [52]. The expression of miRNAs in MM cells can be decreased or increased compared to normal cells and these miRNAs act as oncogenes or tumor suppressors [53]. Kong et al. revealed that the miR-17-5p overexpression enhanced cell growth, colony formation, and cell-cycle progression from G1 to S, in MM cells. Also, miR-17-5p overexpression remarkably diminished the ferroportin (FPN1) expression. In patients with MM, Nrf2 levels were significantly lower, which characterizes patients with a poor prognosis, and knocking out Nrf2 increased miR-17-5p expression in MM cells. Overexpressing Nrf2 increased FPN1 levels, and miR-17-5p overexpression suppressed FPN1 upregulation caused by Nrf2 overexpression. Diminished expression of FPN1 in MM patients is associated with short event-free survival. Actually, Nrf2 directly activated ferroportin expression or inhibited miR-17-5p to target intracellular FPN1, leading to a better understanding that iron metabolism is mediated via the regulation of FPN1. This study detects new roles of both miRNAs and iron-regulatory proteins (IRPs) as post-transcriptional, iron-responsive regulators of iron regulation in MM cell survival and growth [54].

## Neuroblastoma

Neuroblastoma is the highest extracranial solid tumor in children. The heterogeneity of patients varies from low-risk cases, described as better outcomes spontaneously or treated only with surgery, to high-risk cases, defined as consequences of treatment failures.

The activity of the transcription factor that binds to the promoter region of miRNAs is likely to be a determining factor in miRNA expression. The Nrf2 has been documented to regulate the expression of different miRNAs [48, 52].

Recent studies have demonstrated Nrf2 silencing by miR-144, miR-28, and miR-43a in non-neural neuroblastoma cells [55]. Narasimhan et al. evaluated the effects of miRs153/27a/142-5p/144 on Nrf2 in SH-SY5Y cells (derived from neuroblastoma). MiR-144/153/27a/142-5p could inhibit Nrf2 gene expression through 3´ UTR binding and down-modulating Nrf2 expression in SH-SY5Y neuronal cells. MiR-induced inhibition of Nrf2 occurs in a Keap1-independent manner. Mutations in these miR binding sites Nrf2 3´UTR prevent them from interacting, suggesting that Nrf2 is a direct regulatory target of these miRs. Furthermore, Nrf2-dependent redox homeostasis could be regulated in this neuronal system by regulation of levels of the following miRs: miR-144/miR-153/ miR-27a/miR142-5p [56].

## Nasopharyngeal carcinoma (NPC)

NPC is an uncommon malignancy worldwide. Nevertheless, it is endemic in some regions like Southeast Asia, North Africa, and the Arctic [57]. Epidemiological trends revealed a decline in incidence, even in endemic regions, and mortality during the past decade [58]. However, the problem is that more than %70 of NPC patients were diagnosed at advanced stages which decreased their median survival to 3 years. This has made it necessary to improve diagnostic and therapeutic methods for NPC [59, 60].

In 2020, Huang et al. indicated that Raf kinase inhibitor protein (RKIP) exerts its inhibitory effect on NRF/NQO1 through upregulating miR-450b-5p, which binds and inhibits Nrf2 in NPC directly. They demonstrated that RKIP was downregulated and Nrf2 and NQO1 were significantly upregulated in NPC tissues. In addition, they marked that RKIP and miR-450b-5p, as favorable prognostic indicators, were significantly lower in radioresistant NPC tissues, while Nrf2 and NQO1 were significantly higher, which makes them unfavorable prognostic indicators. RKIP and miR-450b-5p downregulation and Nrf2 and NQO1 upregulation were mutually related to malignant pathological parameters, including primary T stages, lymph nodes (N), Metastasis, and TNM stage. They concluded that the RKIP/miR-450b-5p/Nrf2/NQO1 axis has a crucial role in the radioresistance of NPC and could be a potential target for improving NPC clinical treatment [61].

## Hepatocellular carcinoma

Hepatocellular carcinoma (HCC) has been regarded as one of the predominant reasons for cancer-related death. While HCC incidence is increasing globally, its incidence rates are higher in Asia compared to North America and Europe [62, 63]. Infection with hepatitis B virus, alcohol drinking, and a lower intake of calcium and vitamin D are associated with an increased incidence of HCC [64]. Although a wide range of therapeutic choices is used for HCC, chemotherapy is one of the essential approaches to HCC treatment. However, it usually fails because of the inherent resistance of cancerous cells due to DNA damage. The research findings have shown the role of miRNA on a tumor suppressor or oncogenic function mediated differentiation, cell proliferation, and apoptosis. MiRNA- Nrf2 interplay can affect diverse tumor properties, including chemo-resistance [65, 66].

In a recent study, Shi et al. revealed that the over-expression of miR-141 resulted in 5-fluorouracil (5-FU) resistance in HCC cancerous cells by the down-regulation of Keap1 expression. On the other hand, transfection with miR-141 mimics caused miR-141 activation and Keap1 suppression, which led to the re-activation of the Nrf2-dependent antioxidant pathways, inducing 5-FU resistance in hepatocellular carcinoma cells [67]. In a similar study, this same group illustrated that the down-regulation of miR-340 develops cisplatin resistance in HCC cells by up-regulating Nrf2 expression. Interestingly, they also confirmed that miR-340 mimics reduced Nrf2-dependent antioxidant pathway and attenuated chemo-resistance to cisplatin in HCC cells [68]. Wu et al. revealed that the over-expression of lncRNA-NRAL causes the down-regulation of miR-340 and its target gene Nrf2 [69]. In addition, Zhou et al. demonstrated that the miR-144 expression was reduced in HCC cells. This down-regulation of miR-144 leads to over-expression of Nrf2 and HO-1 proteins in Bel-7402 cells resistant to 5-FU. Their results also confirmed that the transfection with miR-144 mimics improves the chemo-sensitivity and enhances apoptosis in Bel-7402 cells resistant to 5-FU through down-regulation of the Nrf2/HO-1 pathway. So, these findings suggest that the targeting of miR-144 can be used as a new strategy in HCC treatment [70]. In another study, Shi et al. proved that down-regulation of miR-340 caused cisplatin chemo-resistance in HepG2 cells through up-regulation of Nrf2. Their results demonstrated that the transfection of HCC cells with miR-340 mimics increased the sensitivity of these cells to cisplatin via attenuating Nrf2 expression. They have represented a different mechanism that miR-340 could potentially increase the sensitivity of HepG2/CDDP cell lines, at least in part, by inhibiting Nrf2 expression [68].

Apigenin, as an Nrf2 activator, is a natural compound that is used in the treatment of different cancers, including lung, colon, ovarian, skin, and prostate cancer. Apigenin is crucial in suppressing proliferation and inducing apoptosis in these cancerous cells. Furthermore, the co-treatment of apigenin with a chemotherapeutic agent improves chemo-sensitivity and apoptosis through modulating β-catenin, PI3K-Akt, and NF-κb signaling pathways [71–73]. Gao et al. illustrated that the treatment of BEL-7402 HCC cells resistant to doxorubicin with apigenin improves chemo-sensitivity to doxorubicin via up-regulation of miR-101 and down-regulation of Nrf2 protein. Therefore, apigenin improves doxorubicin sensitivity by modifying the miR-101/Nrf2 pathway and can potentially be used in combination with doxorubicin to enhance chemotherapy response in HCC [74].

## Lung cancer

Lung cancer is a malignant tumor that has the highest cancer-related death worldwide. With 18% 5-year survival. Surgery resection, radiotherapy, and chemotherapy are the typical strategies used in lung cancer treatment [75, 76]. Cisplatin is an anti-cancer agent that induces apoptosis of lung cancerous cells by inhibiting DNA synthesis. The resistance to chemotherapeutic agents such as cisplatin is the main cause of the low survival rate in lung cancer patients [77]. So, it is necessary to discover molecular mechanisms of chemo-resistance in lung cancer to increase the survival rate of patients.

Dysregulation of miRNAs plays a role in cancer progression [78]. The evidence shows that miRNAs can mediate resistance to radiotherapy and chemotherapy in various cancers, especially lung cancer [79]. On the other hand, Nrf2 is specifically activated in various cancers and can cause chemoresistance, cancer progression, and metastasis [80]. NRF2 has been found to either inhibit or activate the miRNAs expression in various cancers, including lung cancer [81].

Yin et al. indicated that the expression of miR-144 was reduced in lung cancer cells. They confirmed that the transfection of lung cancer cells with miR-144 mimics overcomes cisplatin resistance through attenuating Nrf2 mRNA and protein [82]. LncRNAs have essential roles in biological processes, including chemo-resistance, pairing with RNA polymerase II, influencing the transcription of genes, participating in epigenetic procedures, and modulating splicing through competing with many RNA molecules for binding to miRNAs. For example, LncRNA UCA1 promotes cell growth epigenetically, suppressing p21 and miR-495 expression [83, 84]. Li et al. illustrated that the up-regulation of LncRNA UCA1 and Nrf2 and down-regulation of miR-495 induces cisplatin chemo-resistance in A549 and H460 lung cancer cells. They also demonstrated that the transfection of A549 and H460 cells with miR-490 mimics attenuated the luciferase activity of wild-type UCA1 [85].

Ferroptosis is a new class of cell death that has biochemical and morphological properties different from other types. The production of lipid ROS is increased, but glutathione is decreased in ferroptosis. Erastin has been recognized as a prototype of discovered Ras-selective ferroptotic compounds [86, 87]. In 2020, Gai et al. displayed that the co-delivery of erastin and a nano-delivery system based on folate-modified liposomes (FA-LP) significantly enriched the apoptosis of non-small cell lung cancer (NSCLC) in vitro and in vivo. They also proved that lncRNA-MT1DP reduced Nrf2 expression level and raised the sensitivity of Nrf2-overexpressed NSCLC cells to elastin-induced ferroptosis by stabilizing miR-365a [88].

Ionizing radiation is one of the curative nonsurgical strategies for the treatment of solid tumor cells, especially NSCLC. However, the resistance of NSCLC to radiotherapy restricts the usage of this approach. The targeting of some signaling pathways and microRNAs that cause radiotherapy resistance enhances the outcomes in NSCLC patients [89, 90]. MiR-200c is an important regulator of the EMT process that is downregulated in NSCLC. MiR-200c down-regulation contributes to the increase in cell proliferation, migration, and metastasis. So, therapeutic delivery of miR-200 can improve the radiation efficacy and decrease the metastasis rate of NSCLC cells [91]. Cortez et al. showed that the transfection of A549 lung cancer cells with miR-200c sensitizes tumor cells to cytotoxic effects of radiation by down-regulation of RAD51, PRDX2, GABP/Nrf2, and SESN1 genes and up-regulation of γ-H2AX, and p21 [92].

While IR beams are used in the treatment of cancers, the injury of the lungs is a severe challenge in patients receiving radiation therapy. The lung damage appears 1–6 months after completion of radiation therapy with clinical manifestations such as progressive pneumonitis [93]. Previous studies demonstrated that Nrf2 has a critical function in radioprotection. IR produces free radicals, stabilizes Nrf2, and leads to the activation of anti-inflammation and antioxidant genes, including HO-1, GSTs, and NQO1 by Nrf2 [94]. In another mechanism, BRCA1 binds to cytoplasmic Nrf2 and causes radioprotection by preventing Nrf2 degradation [95]. In the study conducted by Duru et al., IR treatment improved the expression levels of BRCA1 and Nrf2 proteins and facilitated BRCA1 translocation from the nucleus to the cytoplasm, leading to BRCA1-dependent nuclear translocation of Nrf2. They also found that knocking down BRCA1 with siRNA enhances the Keap1 protein. According to their study, miR-140 is a new Nrf2 target gene, and its over-expression suppresses the self-renewal of lung fibroblasts [81].

Arsenic trioxide (ATO) enhances apoptosis by causing oxidative stress and DNA damage in cancerous cells, including prostate, renal, hepatic, acute promyelocytic leukemia (APL), sarcoma, and lung cancers. Previous research revealed that lung cancer cells indicated more resistance to ATO compared to other cancers. So, the investigation of ATO-resistance mechanisms is necessary for the treatment of lung cancer [96–98]. In the study completed by Gu et al., the expression of miR-155 and Nrf2 protein levels were more elevated in A549 ATO-resistant cells compared to non-resistant A549 calls. The transfection of A549 ATO-resistant cells with miR-155 mimic inhibited the colony formation of these cancerous cells by up-regulating Bax and Bcl2 expression. They also proved that the expression of Nrf2, HO-1, and NQO1 was up-regulated in A549 resistant to ATO cells transfected with the miR-155 mimic. This study provides new insight into the role of miR-155 in mediating ATO resistance in lung cancer cells by activating the Nrf2 signaling pathway, increasing cellular antioxidant capacity, and promoting cell survival by modulating cell apoptosis. According to these results, miR-155 can be considered a new therapeutic purpose to combat ATO resistance in lung cancer [98].

## Pancreatic cancer

Pancreatic cancer (PC) is a notoriously fatal malignancy with high mortality, and the 5-year survival rate for PC is 6%. PC is the third most frequent tumor in the USA and the seventh most common cause of cancer-related death worldwide. Familial history, smoking, obesity, alcohol, diabetes mellitus, gender, age, and chronic inflammation are the main risk factors for PC [99, 100]. The diagnosis of PC is a significant concern because of the non-specific symptoms and the proximity of blood vessels in patients with PC. Surgical resection is the only approach for the cure of PC patients. However, the chemotherapy regime FOLFIRONOX is the best palliative strategy in the treatment of PC patients [101, 102].

Recent studies indicated that miRNAs had an essential function in the initiation and biological processes of PC cells. For instance, the down-regulation of miR-373 facilitates the metastasis of PC cells, and the up-regulation of miR-373 improves gemcitabine chemo-sensitivity of PC cells via targeting Cyclin D2 [103]. Another target gene of miR-373, Nrf2, can act as a drug target for antioxidant therapy because it improves antioxidant enzyme expression in response to oxidative stresses. On the other hand, SIRT1 can increase the expression levels of Nrf2 [104, 105]. In 2019, Yin et al. revealed that over-expression of miR-373 significantly decreased the proliferation of pancreatic cancer cells by reducing the expression of SIRT1 in PANC-1 and AsPC-1. The miR-373 up-regulation and SIRT1 down-regulation improve apoptosis-related proteins such as caspase-3, caspase-8, caspase-9, and BAX and suppress BCL-2 and PARP proteins. They also indicated that the expression of Nrf2, PGC-1α, and eNOS proteins was attenuated and increased iNOS protein in miR-373mimics and sh*SIRT1*-transfected PC cells. Altogether, their results suggested that miR-373 up-regulation and SIRT1 down-regulation induced cell death in pancreatic cancerous cells by suppressing the PGC-1α/ Nrf2 pathway, which could highlight miR-373 as a therapeutic target in pancreatic cancer [106].

## Colorectal cancer

Colorectal cancer (CRC) is one of the most commonly occurring cancers in adults globally, and it leads to almost 935,000 cancer-related deaths. The incidence rate and mortality of CRC are rising in developed countries with a Western way of life. Obesity, alcohol, smoking, diabetes mellitus, familial history, inflammatory bowel disease, and excessive consumption of red meat are some of the important risk factors for CRC [107]. Dysregulation of microRNA expression levels is associated with various biological processes, such as metastasis, proliferation, invasion, and chemo-resistance in CRC cells. In addition, microRNAs can be used as markers for early diagnosis, prognosis, predictive high-risk patients, predictive 5-year survival, and recurrence in CRC patients. So, the targeting of these molecules may have a crucial effect on the diagnosis and treatment of CRC [108]. Nowadays, the use of herbal compounds such as curcumin has become necessary in the treatment of cancers. Recent studies proved that curcumin can exert its anti-cancer effects by regulating some microRNAs and signaling pathways in CRC [109]. In the study conducted by Liu et al., they demonstrated that curcumin actives NRF2 gene, which leads to up-regulation of miR-34a and miR-34b/c by binding to their promoters region in CRC cells Overall, the tumor suppressive ability of curcumin is mediated through the activation of the Nrf2/miR-34 pathway, suggesting a new strategy to activate miR-34 genes in cancers as therapeutic targets [110].

## Conclusion

**Fig. 2:**
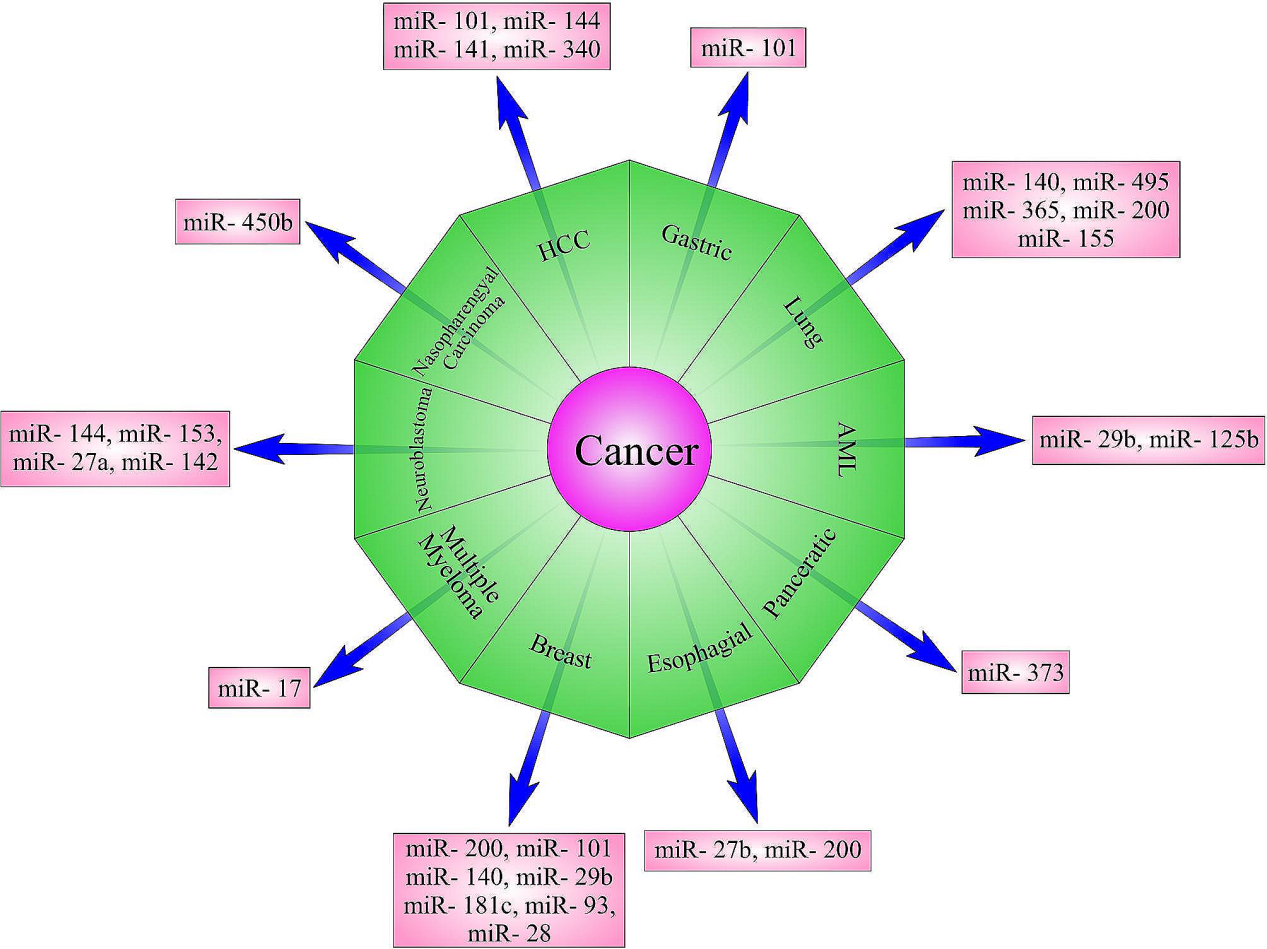
Schematic representation of the interactions between Nrf2 and multiple MicroRNAs in various types of cancer

Today, the focus is on therapies that target signaling pathways and critical molecules in the biology of various cancer cells. MicroRNAs have emerged as key critical regulators of the Nrf2 signaling pathway in various cancers (Fig. 2), offering the potential for therapeutic modulation. Numerous studies across different types of cancer have highlighted the intricate relationship between miRNAs and Nrf2 in cancer development and progression. The dysregulation of miRNAs and their interaction with Nrf2 have been linked to chemoresistance, radioresistance, and cancer development in these malignancies. Furthermore, the identification of NRF2-related miRNA dysregulation in tumor biopsy samples may facilitate the development of personalized clinical treatments related to each patient’s specific tumor characteristics. This approach has the potential to expand the range of available treatments, thus improving 5-year survival rates for patients with gastric, lung, breast, colorectal, esophagus, hepatocellular, pancreas, nasopharyngeal, MM, AML, and neuroblastoma cancers. In addition, targeting these regulatory pathways holds promise for overcoming chemoresistance, inhibiting cancer cell proliferation, and improving clinical outcomes in a wide range of malignancies. However, further researches are needed to elucidate the intricate mechanisms of miRNA-mediated regulation of the Nrf2 pathway and its implications in cancer therapy.

## Data Availability

No datasets were generated or analysed during the current study.

## Abbreviations

MicroRNAs: miRNAs
3'-UTRs: 3'‐untranslated regions
Nrf2: Nuclear factor E2-related factor 2
Rab: Ras‐associated binding
Keap1: Kelch-like ECH associated protein 1
ESCC: Esophageal squamous cell carcinoma
MSA: Methylseleninic acid
GC: Gastric Cancer
Bax: Bcl-2-associated X protein
SAHA: suberoylanilide hydroxamic acid
HDAC: histone deacetylase
MMP: mitochondrial membrane potential
HMEC: human mammary epithelial cells
AML: Acute myeloid leukemia
ARE: antioxidant response element
HSC: hematopoietic stem cells
MM: Multiple Myeloma
BM: bone marrow
PC: plasma cells
NPC: Nasopharyngeal carcinoma
RKIP: Raf kinase inhibitor protein
HCC: Hepatocellular carcinoma
5-FU: 5-fluorouracil
FA-LP: folate-modified liposomes
NSCLC: non-small cell lung cancer
ATO: Arsenic trioxide
APL: acute promyelocytic leukemia

## References

[1] Sung H, Ferlay J, Siegel RL, Laversanne M, Soerjomataram I, Jemal A, Bray F (2021). Global cancer statistics 2020: GLOBOCAN estimates of incidence and mortality worldwide for 36 cancers in 185 countries. Cancer J Clin.

[2] Pouremamali F, Pouremamali A, Dadashpour M, Soozangar N, Jeddi F (2022). An update of Nrf2 activators and inhibitors in cancer prevention/promotion. Cell Communication Signal.

[3] Jung B-J, Yoo H-S, Shin S, Park Y-J, Jeon S-M (2018). Dysregulation of NRF2 in cancer: from molecular mechanisms to therapeutic opportunities. Biomolecules Ther.

[4] Wu S, Lu H, Bai Y (2019). Nrf2 in cancers: a double-edged sword. Cancer Med.

[5] O’Brien J, Hayder H, Zayed Y, Peng C (2018). Overview of microRNA biogenesis, mechanisms of actions, and circulation. Front Endocrinol.

[6] Ji S, Yang Z, Gozali L, Kenney T, Kocabas A, Jinsook Park C, Hynes M (2021). Distinct expression of select and transcriptome-wide isolated 3’UTRs suggests critical roles in development and transition states. PLoS ONE.

[7] Ashrafizadeh M, Ahmadi Z, Samarghandian S, Mohammadinejad R, Yaribeygi H, Sathyapalan T, Sahebkar A (2020). MicroRNA-mediated regulation of Nrf2 signaling pathway: implications in disease therapy and protection against oxidative stress. Life Sci.

[8] Yang M, Yao Y, Eades G, Zhang Y, Zhou Q (2011). MiR-28 regulates Nrf2 expression through a Keap1-independent mechanism. Breast Cancer Res Treat.

[9] Quiles JM, Pepin ME, Sunny S, Shelar SB, Challa AK, Dalley B, Hoidal JR, Pogwizd SM, Wende AR, Rajasekaran NS (2021). Identification of Nrf2-responsive microRNA networks as putative mediators of myocardial reductive stress. Sci Rep.

[10] Jeddi F, Soozangar N, Sadeghi MR, Somi MH, Samadi N (2017). Contradictory roles of Nrf2/Keap1 signaling pathway in cancer prevention/promotion and chemoresistance. DNA Repair.

[11] Ayers D, Baron B, Hunter T. miRNA influences in NRF2 pathway interactions within cancer models. *Journal of Nucleic Acids* 2015, 2015. 10.1155/2015/143636 PMC4546755 26345522

[12] Zhang C, Shu L, Kong A-NT (2015). MicroRNAs: new players in cancer prevention targeting Nrf2, oxidative stress and inflammatory pathways. Curr Pharmacol Rep.

[13] Abnet CC, Arnold M, Wei W-Q (2018). Epidemiology of esophageal squamous cell carcinoma. Gastroenterology.

[14] Hemmatzadeh M, Mohammadi H, Karimi M, Musavishenas MH, Baradaran B (2016). Differential role of microRNAs in the pathogenesis and treatment of esophageal cancer. Biomed Pharmacother.

[15] Han M, Li N, Li F, Wang H, Ma L (2020). MiR-27b-3p exerts tumor suppressor effects in esophageal squamous cell carcinoma by targeting Nrf2. Hum Cell.

[16] Liu M, Hu C, Xu Q, Chen L, Ma K, Xu N, Zhu H. Methylseleninic acid activates Keap1/Nrf2 pathway via up-regulating miR-200a in human oesophageal squamous cell carcinoma cells. Biosci Rep 2015, 35(5). 10.1042/BSR20150092 PMC4613709 26341629

[17] Fatehi-Agdam M, Vatankhah MA, Panahizadeh R, Jeddi F, Najafzadeh N (2021). Efficacy of metformin and chemotherapeutic agents on the inhibition of colony formation and Shh/Gli1 pathway: Metformin/docetaxel versus metformin/5-fluorouracil. Drug Res.

[18] Khakbaz P, Panahizadeh R, Vatankhah MA, Najafzadeh N (2021). Allicin reduces 5-fluorouracil-resistance in gastric Cancer cells through modulating MDR1, DKK1, and WNT5A expression. Drug Res.

[19] Chorley BN, Campbell MR, Wang X, Karaca M, Sambandan D, Bangura F, Xue P, Pi J, Kleeberger SR, Bell DA (2012). Identification of novel NRF2-regulated genes by ChIP-Seq: influence on retinoid X receptor alpha. Nucleic Acids Res.

[20] Singh A, Happel C, Manna SK, Acquaah-Mensah G, Carrerero J, Kumar S, Nasipuri P, Krausz KW, Wakabayashi N, Dewi R (2013). Transcription factor NRF2 regulates miR-1 and miR-206 to drive tumorigenesis. J Clin Investig.

[21] Shrestha S, Hsu SD, Huang WY, Huang HY, Chen W, Weng SL, Huang HD (2014). A systematic review of microRNA expression profiling studies in human gastric cancer. Cancer Med.

[22] Jeddi F, Alipour S, Najafzadeh N, Dadashpour M, Pouremamali F, Sadeghi MR, Samadi N, Soozangar N, Khamaneh AM (2019). Reduced levels of miR–28 and miR–200a act as predictor biomarkers of aggressive clinicopathological characteristics in gastric cancer patients. Galen Med J.

[23] Dong X, Zhang Y, Shang X, Zeng Y (2019). Effects of miR-101 on the proliferation and apoptosis of gastric mucosal epithelial cells via Nrf2/ARE signaling pathway. Eur Rev Med Pharmacol Sci.

[24] Schnitt S. Pathology of invasive breast cancer. Dis Breast 2000.

[25] Saw CL-L, Wu Q, Kong A-N (2010). Anti-cancer and potential chemopreventive actions of ginseng by activating Nrf2 (NFE2L2) anti-oxidative stress/anti-inflammatory pathways. Chin Med.

[26] Ohtsuji M, Katsuoka F, Kobayashi A, Aburatani H, Hayes JD, Yamamoto M (2008). Nrf1 and Nrf2 play distinct roles in activation of antioxidant response element-dependent genes. J Biol Chem.

[27] Zimmerman AL, Wu S (2011). MicroRNAs, cancer and cancer stem cells. Cancer Lett.

[28] Kaspar JW, Niture SK, Jaiswal AK (2009). Nrf2: INrf2 (Keap1) signaling in oxidative stress. Free Radic Biol Med.

[29] Eades G, Yang M, Yao Y, Zhang Y, Zhou Q (2011). miR-200a regulates Nrf2 activation by targeting Keap1 mRNA in breast cancer cells. J Biol Chem.

[30] Sarkar S, Abujamra AL, Loew JE, Forman LW, Perrine SP, Faller DV (2011). Histone deacetylase inhibitors reverse CpG methylation by regulating DNMT1 through ERK signaling. Anticancer Res.

[31] Semenza GL (2016). The hypoxic tumor microenvironment: a driving force for breast cancer progression. Biochim et Biophys Acta (BBA)-Molecular Cell Res.

[32] Bell EL, Klimova TA, Eisenbart J, Schumacker PT, Chandel NS (2007). Mitochondrial reactive oxygen species trigger hypoxia-inducible factor-dependent extension of the replicative life span during hypoxia. Mol Cell Biol.

[33] Toth RK, Warfel NA (2017). Strange bedfellows: nuclear factor, erythroid 2-like 2 (Nrf2) and hypoxia-inducible factor 1 (HIF-1) in tumor hypoxia. Antioxidants.

[34] Zhao R, Feng J, He G (2016). Hypoxia increases Nrf2-induced HO-1 expression via the PI3K/Akt pathway. Front Bioscience-Landmark.

[35] Mahajan M, Sitasawad S (2021). Mir-140-5p attenuates hypoxia-induced breast cancer progression by targeting nrf2/ho-1 axis in a keap1-independent mechanism. Cells.

[36] De Blasio A, Di Fiore R, Pratelli G, Drago-Ferrante R, Saliba C, Baldacchino S, Grech G, Scerri C, Vento R, Tesoriere G (2020). A loop involving NRF2, miR‐29b‐1‐5p and AKT, regulates cell fate of MDA‐MB‐231 triple‐negative breast cancer cells. J Cell Physiol.

[37] Yi J, Huang W, Wen Y, Yi Y (2019). Effect of miR-101 on proliferation and oxidative stress-induced apoptosis of breast cancer cells via Nrf2 signaling pathway. Eur Rev Med Pharmacol Sci.

[38] Lee S, Hallis SP, Jung K-A, Ryu D, Kwak M-K (2019). Impairment of HIF-1α-mediated metabolic adaption by NRF2-silencing in breast cancer cells. Redox Biol.

[39] Singh B, Mense SM, Remotti F, Liu X, Bhat HK (2009). Antioxidant butylated hydroxyanisole inhibits estrogen-induced breast carcinogenesis in female ACI rats. J Biochem Mol Toxicol.

[40] Mense SM, Remotti F, Bhan A, Singh B, El-Tamer M, Hei TK, Bhat HK (2008). Estrogen-induced breast cancer: alterations in breast morphology and oxidative stress as a function of estrogen exposure. Toxicol Appl Pharmcol.

[41] Singh B, Ronghe AM, Chatterjee A, Bhat NK, Bhat HK (2013). MicroRNA-93 regulates NRF2 expression and is associated with breast carcinogenesis. Carcinogenesis.

[42] Ramos-Gomez M, Kwak M-K, Dolan P, Itoh K, Yamamoto M, Talalay P, Kensler TW (2001). Sensitivity to carcinogenesis is increased and chemoprotective efficacy of enzyme inducers is lost in nrf2 transcription factor-deficient mice. Proc Natl Acad Sci.

[43] Filipowicz W, Bhattacharyya SN, Sonenberg N (2008). Mechanisms of post-transcriptional regulation by microRNAs: are the answers in sight?. Nat Rev Genet.

[44] Yang M, Yao Y, Eades G, Zhang Y, Zhou Q (2011). MiR-28 regulates Nrf2 expression through a Keap1-independent mechanism. Breast Cancer Res Treat.

[45] Alam J, Stewart D, Touchard C, Boinapally S, Choi AM, Cook JL (1999). Nrf2, a Cap’n’Collar transcription factor, regulates induction of the heme oxygenase-1 gene. J Biol Chem.

[46] Rushworth SA, Zaitseva L, Murray MY, Shah NM, Bowles KM, MacEwan DJ (2012). The high Nrf2 expression in human acute myeloid leukemia is driven by NF-κB and underlies its chemo-resistance. Blood J Am Soc Hematol.

[47] Garzon R, Marcucci G, Croce CM (2010). Targeting microRNAs in cancer: rationale, strategies and challenges. Nat Rev Drug Discovery.

[48] Shah N, Zaitseva L, Bowles K, MacEwan D, Rushworth S (2015). NRF2-driven miR-125B1 and miR-29B1 transcriptional regulation controls a novel anti-apoptotic miRNA regulatory network for AML survival. Cell Death Differ.

[49] Bousquet M, Harris MH, Zhou B, Lodish HF (2010). MicroRNA miR-125b causes leukemia. Proc Natl Acad Sci.

[50] Pinto V, Bergantim R, Caires HR, Seca H, Guimarães JE, Vasconcelos MH (2020). Multiple myeloma: available therapies and causes of drug resistance. Cancers.

[51] Anderson KC, Carrasco RD (2011). Pathogenesis of myeloma. Annu Rev Pathol.

[52] Handa H, Murakami Y, Ishihara R, Kimura-Masuda K, Masuda Y (2019). The role and function of microRNA in the pathogenesis of multiple myeloma. Cancers.

[53] Desantis V, Saltarella I, Lamanuzzi A, Melaccio A, Solimando AG, Mariggiò MA, Racanelli V, Paradiso A, Vacca A, Frassanito MA (2020). MicroRNAs-based nano-strategies as new therapeutic approach in multiple myeloma to overcome disease progression and drug resistance. Int J Mol Sci.

[54] Kong Y, Hu L, Lu K, Wang Y, Xie Y, Gao L, Yang G, Xie B, He W, Chen G (2019). Ferroportin downregulation promotes cell proliferation by modulating the Nrf2–miR-17-5p axis in multiple myeloma. Cell Death Dis.

[55] Breving K, Esquela-Kerscher A (2010). The complexities of microRNA regulation: mirandering around the rules. Int J Biochem Cell Biol.

[56] Narasimhan M, Patel D, Vedpathak D, Rathinam M, Henderson G, Mahimainathan L (2012). Identification of novel microRNAs in post-transcriptional control of Nrf2 expression and redox homeostasis in neuronal, SH-SY5Y cells. PLoS ONE.

[57] Wu L, Li C, Pan L (2018). Nasopharyngeal carcinoma: a review of current updates. Exp Ther Med.

[58] Chua MLK, Wee JTS, Hui EP, Chan ATC (2016). Nasopharyngeal carcinoma. Lancet.

[59] Tang LQ, Li CF, Li J, Chen WH, Chen QY, Yuan LX, Lai XP, He Y, Xu YX, Hu DP et al. Establishment and validation of Prognostic Nomograms for endemic nasopharyngeal carcinoma. J Natl Cancer Inst 2016, 108(1). 10.1093/jnci/djv291 26467665

[60] Chua DT, Sham JS, Kwong DL, Au GK (2003). Treatment outcome after radiotherapy alone for patients with stage I-II nasopharyngeal carcinoma. Cancer.

[61] Huang W, Shi G, Yong Z, Li J, Qiu J, Cao Y, Zhao Y, Yuan L (2020). Downregulation of RKIP promotes radioresistance of nasopharyngeal carcinoma by activating NRF2/NQO1 axis via downregulating miR-450b-5p. Cell Death Dis.

[62] Ferlay J, Soerjomataram I, Dikshit R, Eser S, Mathers C, Rebelo M, Parkin DM, Forman D, Bray F (2015). Cancer incidence and mortality worldwide: sources, methods and major patterns in GLOBOCAN 2012. Int J Cancer.

[63] Ikeda M, Mitsunaga S, Ohno I, Hashimoto Y, Takahashi H, Watanabe K, Umemoto K, Okusaka T (2015). Systemic chemotherapy for advanced hepatocellular carcinoma: past, present, and future. Diseases.

[64] Sun Y, Ma W, Yang Y, He M, Li A, Bai L, Yu B, Yu Z (2019). Cancer nanotechnology: enhancing tumor cell response to chemotherapy for hepatocellular carcinoma therapy. Asian J Pharm Sci.

[65] Carta A, Chetcuti R, Ayers D. An introspective update on the influence of miRNAs in breast carcinoma and neuroblastoma chemoresistance. *Genetics Research International* 2014, 2014. 10.1155/2014/743050 PMC4273469 25548681

[66] Li Y, Ahmad A, Kong D, Bao B, Sarkar FH (2013). Targeting microRNAs for personalized cancer therapy. Med Principles Pract.

[67] Shi L, Wu L, Chen Z, Yang J, Chen X, Yu F, Zheng F, Lin X (2015). MiR-141 activates Nrf2-dependent antioxidant pathway via down-regulating the expression of Keap1 conferring the resistance of hepatocellular carcinoma cells to 5-fluorouracil. Cell Physiol Biochem.

[68] Shi L, Chen Z-G, Wu L-l, Zheng J-J, Yang J-R, Chen X-F, Chen Z-Q, Liu C-L, Chi S-Y, Zheng J-Y (2015). miR-340 reverses cisplatin resistance of hepatocellular carcinoma cell lines by targeting Nrf2-dependent antioxidant pathway. Asian Pac J Cancer Prev.

[69] Wu L-l, Cai W-p, Lei X, Shi K-q, Lin X-y, Shi L (2019). NRAL mediates cisplatin resistance in hepatocellular carcinoma via miR-340-5p/Nrf2 axis. J Cell Communication Signal.

[70] Zhou S, Ye W, Zhang Y, Yu D, Shao Q, Liang J, Zhang M (2016). miR-144 reverses chemoresistance of hepatocellular carcinoma cell lines by targeting Nrf2-dependent antioxidant pathway. Am J Translational Res.

[71] Sung B, Chung HY, Kim ND (2016). Role of apigenin in cancer prevention via the induction of apoptosis and autophagy. J cancer Prev.

[72] Fang J, Xia C, Cao Z, Zheng JZ, Reed E, Jiang B-H (2005). Apigenin inhibits VEGF and HIF-1 expression via PI3K/AKT/p70S6K1 and HDM2/p53 pathways. FASEB J.

[73] Tong X, Pelling C (2013). Targeting the PI3K/Akt/mTOR axis by apigenin for cancer prevention. Anti-Cancer Agents Med Chem (Formerly Curr Med Chemistry-Anti-Cancer Agents).

[74] Gao A-M, Zhang X-Y, Ke Z-P (2017). Apigenin sensitizes BEL-7402/ADM cells to doxorubicin through inhibiting miR-101/Nrf2 pathway. Oncotarget.

[75] Jones GS, Baldwin DR (2018). Recent advances in the management of lung cancer. Clin Med.

[76] Waqar SN, Morgensztern D (2017). Treatment advances in small cell lung cancer (SCLC). Pharmacol Ther.

[77] Barr MP, Gray SG, Hoffmann AC, Hilger RA, Thomale J, O’Flaherty JD, Fennell DA, Richard D, O’Leary JJ, O’Byrne KJ (2013). Generation and characterisation of cisplatin-resistant non-small cell lung cancer cell lines displaying a stem-like signature. PLoS ONE.

[78] Croce CM (2009). Causes and consequences of microRNA dysregulation in cancer. Nat Rev Genet.

[79] MacDonagh L, Gray SG, Finn SP, Cuffe S, O’Byrne KJ, Barr MP (2015). The emerging role of microRNAs in resistance to lung cancer treatments. Cancer Treat Rev.

[80] Gu S, Lai Y, Chen H, Liu Y, Zhang Z (2017). miR-155 mediates arsenic trioxide resistance by activating Nrf2 and suppressing apoptosis in lung cancer cells. Sci Rep.

[81] Duru N, Gernapudi R, Zhang Y, Yao Y, Lo P-K, Wolfson B, Zhou Q (2015). NRF2/miR-140 signaling confers radioprotection to human lung fibroblasts. Cancer Lett.

[82] Yin Y, Liu H, Xu J, Shi D, Zhai L, Liu B, Wang L, Liu G, Qin J (2018). miR–144–3p regulates the resistance of lung cancer to cisplatin by targeting Nrf2. Oncol Rep.

[83] Managadze D, Rogozin IB, Chernikova D, Shabalina SA, Koonin EV (2011). Negative correlation between expression level and evolutionary rate of long intergenic noncoding RNAs. Genome Biol Evol.

[84] Mercer TR, Dinger ME, Sunkin SM, Mehler MF, Mattick JS. Specific expression of long noncoding RNAs in the mouse brain. *Proceedings of the National Academy of Sciences* 2008, 105(2):716–721. 10.1073/pnas.0706729105 PMC2206602 18184812

[85] Li C, Fan K, Qu Y, Zhai W, Huang A, Sun X, Xing S (2020). Deregulation of UCA1 expression may be involved in the development of chemoresistance to cisplatin in the treatment of non-small‐cell lung cancer via regulating the signaling pathway of microRNA‐495/NRF2. J Cell Physiol.

[86] Conrad M, Kagan VE, Bayir H, Pagnussat GC, Head B, Traber MG, Stockwell BR (2018). Regulation of lipid peroxidation and ferroptosis in diverse species. Genes Dev.

[87] Yu Y, Xie Y, Cao L, Yang L, Yang M, Lotze MT, Zeh HJ, Kang R, Tang D (2015). The ferroptosis inducer erastin enhances sensitivity of acute myeloid leukemia cells to chemotherapeutic agents. Mol Cell Oncol.

[88] Gai C, Liu C, Wu X, Yu M, Zheng J, Zhang W, Lv S, Li W (2020). MT1DP loaded by folate-modified liposomes sensitizes erastin-induced ferroptosis via regulating miR-365a-3p/NRF2 axis in non-small cell lung cancer cells. Cell Death Dis.

[89] Chinnaiyan P, Huang S, Vallabhaneni G, Armstrong E, Varambally S, Tomlins SA, Chinnaiyan AM, Harari PM (2005). Mechanisms of enhanced radiation response following epidermal growth factor receptor signaling inhibition by erlotinib (Tarceva). Cancer Res.

[90] Welsh JW, Komaki R, Amini A, Munsell MF, Unger W, Allen PK, Chang JY, Wefel JS, McGovern SL, Garland LL (2013). Phase II trial of erlotinib plus concurrent whole-brain radiation therapy for patients with brain metastases from non–small-cell lung cancer. J Clin Oncol.

[91] Pecot CV, Rupaimoole R, Yang D, Akbani R, Ivan C, Lu C, Wu S, Han H-D, Shah MY, Rodriguez-Aguayo C (2013). Tumour angiogenesis regulation by the miR-200 family. Nat Commun.

[92] Cortez MA, Valdecanas D, Zhang X, Zhan Y, Bhardwaj V, Calin GA, Komaki R, Giri DK, Quini CC, Wolfe T (2014). Therapeutic delivery of miR-200c enhances radiosensitivity in lung cancer. Mol Ther.

[93] Ghafoori P, Marks LB, Zeljko Vujaskovic M, Kelsey CR (2008). Radiation-induced lung injury: assessment, management, and prevention. Oncology.

[94] Ray PD, Huang B-W, Tsuji Y (2012). Reactive oxygen species (ROS) homeostasis and redox regulation in cellular signaling. Cell Signal.

[95] Gorrini C, Baniasadi PS, Harris IS, Silvester J, Inoue S, Snow B, Joshi PA, Wakeham A, Molyneux SD, Martin B (2013). BRCA1 interacts with Nrf2 to regulate antioxidant signaling and cell survival. J Exp Med.

[96] Liu J-X, Zhou G-B, Chen S-J, Chen Z (2012). Arsenic compounds: revived ancient remedies in the fight against human malignancies. Curr Opin Chem Biol.

[97] Fan X-Y, Chen X-Y, Liu Y-J, Zhong H-M, Jiang F-L, Liu Y (2016). Oxidative stress-mediated intrinsic apoptosis in human promyelocytic leukemia HL-60 cells induced by organic arsenicals. Sci Rep.

[98] Gu S, Lai Y, Chen H, Liu Y, Zhang Z (2017). miR-155 mediates arsenic trioxide resistance by activating Nrf2 and suppressing apoptosis in lung cancer cells. Sci Rep.

[99] Bray F, Ferlay J, Soerjomataram I, Siegel RL, Torre LA, Jemal A (2018). Global cancer statistics 2018: GLOBOCAN estimates of incidence and mortality worldwide for 36 cancers in 185 countries. Cancer J Clin.

[100] Rawla P, Sunkara T, Gaduputi V (2019). Epidemiology of pancreatic cancer: global trends, etiology and risk factors. World J Oncol.

[101] Canto MI, Harinck F, Hruban RH, Offerhaus GJ, Poley J-W, Kamel I, Nio Y, Schulick RS, Bassi C, Kluijt I (2013). International Cancer of the pancreas Screening (CAPS) Consortium summit on the management of patients with increased risk for familial pancreatic cancer. Gut.

[102] Conroy T, Desseigne F, Ychou M, Bouché O, Guimbaud R, Bécouarn Y, Adenis A, Raoul J-L, de la Gourgou-Bourgade S (2011). Fouchardière C: FOLFIRINOX versus gemcitabine for metastatic pancreatic cancer. N Engl J Med.

[103] Hu W, Liu Q, Pan J, Sui Z (2018). MiR-373-3p enhances the chemosensitivity of gemcitabine through cell cycle pathway by targeting CCND2 in pancreatic carcinoma cells. Biomed Pharmacother.

[104] Kubo Y, Wruck CJ, Fragoulis A, Drescher W, Pape HC, Lichte P, Fischer H, Tohidnezhad M, Hildebrand F, Pufe T (2019). Role of Nrf2 in fracture healing: clinical aspects of oxidative stress. Calcif Tissue Int.

[105] Huang K, Huang J, Xie X, Wang S, Chen C, Shen X, Liu P, Huang H (2013). Sirt1 resists advanced glycation end products-induced expressions of fibronectin and TGF-β1 by activating the Nrf2/ARE pathway in glomerular mesangial cells. Free Radic Biol Med.

[106] Yin Q-H, Zhou Y, Li Z-Y (2021). miR-373 suppresses cell proliferation and apoptosis via regulation of SIRT1/PGC-1α/NRF2 axis in pancreatic cancer. Cell J (Yakhteh).

[107] Sawicki T, Ruszkowska M, Danielewicz A, Niedźwiedzka E, Arłukowicz T, Przybyłowicz KE (2021). A review of colorectal cancer in terms of epidemiology, risk factors, development, symptoms and diagnosis. Cancers.

[108] Zhao P, Cheng J, Li B, Nie D, Wang H, Li C, Gui S, Zhang Y (2021). LncRNA PCAT6 regulates the progression of pituitary adenomas by regulating the miR-139-3p/BRD4 axis. Cancer Cell Int.

[109] Li J, Chai R, Chen Y, Zhao S, Bian Y, Wang X (2022). Curcumin targeting non-coding RNAs in colorectal cancer: therapeutic and biomarker implications. Biomolecules.

[110] Liu C, Rokavec M, Huang Z, Hermeking H (2023). Curcumin activates a ROS/KEAP1/NRF2/miR-34a/b/c cascade to suppress colorectal cancer metastasis. Cell Death Differ.

